# Photo-Irradiated Biosynthesis of Silver Nanoparticles Using Edible Mushroom *Pleurotus florida* and Their Antibacterial Activity Studies

**DOI:** 10.1155/2011/650979

**Published:** 2011-12-10

**Authors:** Ravishankar Bhat, Raghunandan Deshpande, Sharanabasava V. Ganachari, Do Sung Huh, A. Venkataraman

**Affiliations:** ^1^Materials Chemistry Laboratory, Department of Material Science, Gulbarga University, Gulbarga 585106, Karnataka, India; ^2^H.K.E.'s Matoshree Taradevi Rampure Institute of Pharmaceutical Sciences, Sedam Road, Gulbarga 585105, Karnataka, India; ^3^Department of Chemistry, Inje University, Kimhae, Kyungnam 621749, Republic of Korea

## Abstract

This is a report on photo-irradiated extracellular synthesis of silver nanoparticles using the aqueous extract of edible oyster mushroom (*Pleurotus florida*) as a reducing agent. The appearance, size, and shape of the silver nanoparticles are understood by UV-visible spectroscopy, field emission scanning electron microscopy, transmission electron microscopy, and atomic force microscopy. The X-ray diffraction studies, energy dispersive X-ray analysis indicate that particles are crystalline in nature. Fourier transform infrared spectroscopy analysis revealed that the nanoparticles are covered with biomoieties on their surface. As can be seen from our studies, the biofunctionalized silver nanoparticles thus produced have shown admirable antimicrobial effects, and the synthetic procedure involved is eco-friendly and simple, and hence high range production of the same can be considered for using them in many pharmaceutical applications.

## 1. Introduction

**Figure 1:**
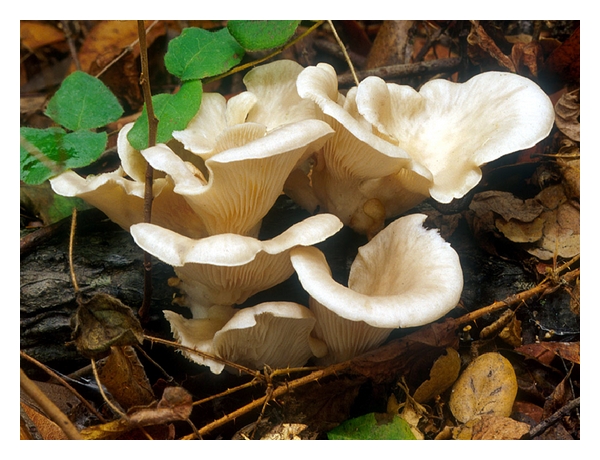
Image of mushroom *Pleurotus florida*.

Nanotechnology is a rapidly growing field which has led to promising revolutionary applications in medical and engineering in terms of their efficacy, safety and economy. Nanobiotechnology is an offspring of nanotechnology that has emerged at the interface of nanotechnology and biology. Nanoparticles smeared with chemicals or biomoieties are gaining interest in the field of nanodrug delivery systems which have specific and localized applications without harming the cells of the surrounding areas of the body organs. Hence, there is a need to develop green chemistry approaches in the synthesis for the nanomaterials [1]. In this aspect, synthetic methods based on naturally occurring biomaterials offer better alternatives [2]. The bioroutes for the synthesis of the nanoparticles include employing microorganisms such as *Pseudomonas stutzeri* [3], *Plectonema boryanum* UTEX485 [4], *Verticillium sp.* [5], *Fusarium oxysporium *[6], *Fusarium semitectum* [7], MKY3 (yeast) [8], *Thermomonospora sp.* [9], and also different plants like *Avena sativa* [10], *Aloe vera *[11], *Azadiracta indica* (neem) [12], *Psdium guajava *(Guava plant) [13], and so forth. Few mushrooms (spore-bearing fruiting body of fungus) are also used for this purpose, namely, *Volvariella volvacea* [14], *Pleurotus sajor* [15]. In this work, we have used extract of edible mushroom *Pleurotus florida *which is also known as Oyster mushroom (Figure 1) for the synthesis of biofunctionalized silver nanoparticles (AgNPs) as this mushroom is known for its medicinal property [16] as an antioxidant and antitumor agent [17].

Since antiquity silver in different forms has been extensively used as a medicine for curing diseases and promote wound healing [18]. AgNPs have high specific area than their volume, which will lead to excellent antimicrobial activity as compared with bulk Ag metal [19]. Antimicrobial properties of AgNPs are well demonstrated against both bacteria [20] and viruses [21]. This is because of close attachment of the nanoparticles surface with microbial cells or viruses, and hence its antimicrobial property is size dependent. AgNPs are currently used as an active drug in targeted drug delivery [22], gene delivery [23], and artificial implants [24] and as a diagnostic agent for imaging and sensing in different diseases at their early stages. Owing to their mutation-resistant antimicrobial activity, they are being used in different pharmaceutical formulations as antibacterial clothing [25], burn ointments [26], and coating for medical devices [27]. As studies of AgNPs improved, several medical applications have been developed to prevent the onset of infection and promote faster wound healing [28]. In this project, we have made an effort to develop eco-friendly method for the synthesis of AgNPs and studied using different sophisticated instruments for the characterization of the particles.

AgNPs can be successfully synthesized by using a variety of synthesis methods like heating technique [29], laser irradiation [30], ionizing radiation [31], and radiolysis [32]. The chemical and physical methods for nanomaterial manufacturing are found to be quite expensive and nonfriendly to environment.

The upcoming bottom-up self-assembly biosynthesis procedures are maceration, heating, or exposure to laser for the production of AgNPs [33], which are simple, eco-friendly, cost effective, and also time saving. In line with this, we have synthesized stable biofunctionalized AgNPs using edible mushroom *P. florida *extract and silver salt (AgNO_3_) with the aid of photo irradiation, that is, exposing the reaction mixture to direct sunlight. These AgNPs have been tested for the antimicrobial property using stock cultures of *Staphylococcus aureus, Salmonella typhi, Providencia alcalifaciens,* and* Proteus mirabilis *bacteria's and MIC; changes in bacterial count are measured and reported.

## 2. Experimental

### 2.1. Materials

Fresh mushrooms *Pleurotus florida *(Oyster mushroom) are obtained from Forest College, Sirsi, North Canara district. Silver Nitrate (AgNO_3_) A.R grade is procured from Hi-Media Laboratories.

### 2.2. Methodology

The 5 gm fresh mushrooms washed repeatedly with distilled water to remove any organic impurities present on it. The cleaned mushrooms are then crushed to small pieces with a sterilized knife. The small pieces of mushrooms are then taken in to 1 L beaker containing 500 mL double distilled water and thoroughly stirred for about half an hour. The same is kept for overnight and filtered twice with Waltman filter paper no. 42. The resultant filtrate is the extract of mushroom used for the reduction of Ag^+^ to Ag^0^. The mushroom extract is treated with aq. 10^−3^ M AgNO_3_ solution and exposed to bright sunlight; the change of color takes place within few minutes from colourless to reddish brown colour, whereas no color change is observed in the solution kept in dark room.

### 2.3. Antimicrobial Activity

The nutrient broth is prepared using Peptone 10 g; NaCl 10 g and yeast extract 5 g, agar 20 g in 1 L of distilled water. Initially, the stock cultures of *Staphylococcus aureus, Salmonella typhi, Providencia alcalifaciens,* and* Proteus mirabilis* were revived by inoculating the broth media and grown at 37°C for 18 h. The media was autoclaved and cooled to ~55°C. The required volume of test samples containing functionalized AgNPs (produced as above) at different concentrations (viz., 2.5, 5, 10, 20 *μ*g/mL) were added and mixed well. The media was poured into the preautoclaved petri dishes. The 104 CFU/mL culture was inoculated and grown at 37°C for 24 h. The control plate (without sample) and standard plate (with standard sample) were also studied for comparison purpose.

### 2.4. Characterization

The formation of AgNPs is verified by using UV-vis 5704SS ECIL spectrophotometer operated at 1 nm resolution with an optical length of 10 mm. UV-vis analysis of the reaction mixture was observed for a period of 300 sec. For the study of crystallinity, X-ray diffraction (XRD) studies were conducted using Siemens X-ray diffractrometer (Japan), operated at 30 kV and 20 mA current with CuK*α* (I = 1.54 A°). Films of colloidal form AgNPs were tested by drop coating on Si (III) substrates, and data were recorded. The images of Atomic Force Microscope (AFM) were collected under ambient conditions on a Veeco-Innova scanning probe microscope, etched Si-nano probe tips (RTESPA-M) were used for the same. The transmission electron microscopy (TEM) images were obtained using Technai-20 Philips instrument operated at 190 Kev. Samples for this analysis were prepared by coating the aqueous AgNPs on carbon-coated copper grids, after 5 min the extra solution was removed using blotting paper, and then the films of the grids are exposed to IR light for drying.

 For FTIR studies, the powder sample of AgNPs was prepared by centrifuging the synthesized AgNP solution at 10,000 rpm for 20 min. The solid residue obtained is then washed with deionized water to remove any unattached biological moieties to the surface of the nanoparticles, which are not responsible for biofunctionalization and capping. The resultant residue is then dried completely, and the powder obtained is used for FTIR measurements carried out on a Perkin-Elmer spectrum one, instrument at a spectral resolution of 4 cm^−1^ in KBr pellets.

## 3. Results and Discussion

**Figure 2:**
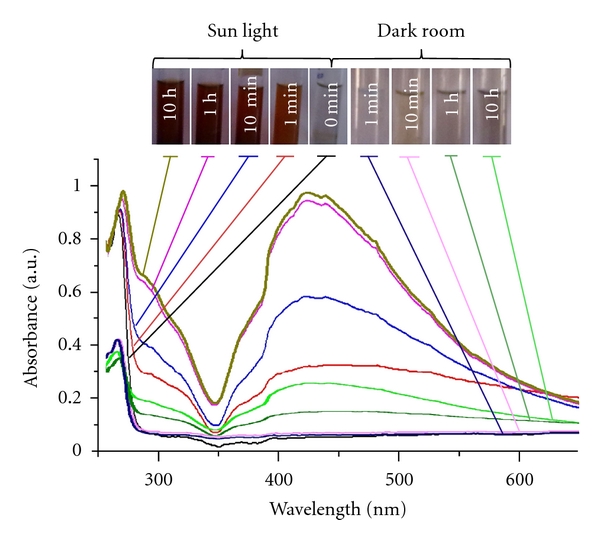
UV-vis spectra indicating the photo-irradiated AgNP synthesis recorded as a function of time. The change in color of the reaction mixture and its respective surface plasmon resonance is shown with the lines.

The change in color of the reaction mixture from colorless to reddish brown is observed within minutes of photo irradiation. AgNP shows light brown color in water which is a clear indication for the formation of AgNPs. This color arises due to excitation of surface plasmon vibrations in metal nanoparticles [34]. This is observed in UV-visible spectra. Absorbance intensity of reddish brown color increases steadily as a function of reaction time.  Figure 2 shows the UV-visible spectra recorded as absorbance versus reaction time during the synthesis of AgNPs from aq. 10^−3^ M AgNO_3_ and extracellular filtrate of the mushroom *Pleurotus florida* biomass mixture. It is observed that the band corresponding to surface plasmon resonance occurs at 435 nm which clearly indicates the formation of AgNPs in the reaction mixture [34]. For comparison purpose, 20 mL of aqueous 10^−3^ M AgNO_3_ solution was exposed to sunlight for the blank analysis and subjected to UV-vis study. It is observed that there is no colour change in aq. AgNO_3_ solution since nanoparticles are not formed.

**Figure 3:**
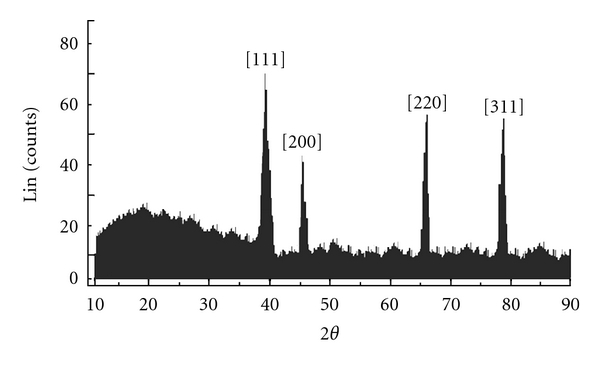
XRD pattern of crystalline AgNPs.

**Figure 4:**
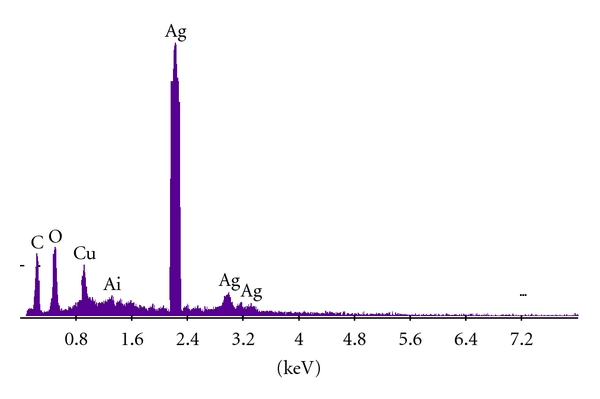
Energy dispersive X-ray spectrum (EDAX) of metallic AgNP.

**Figure 5:**
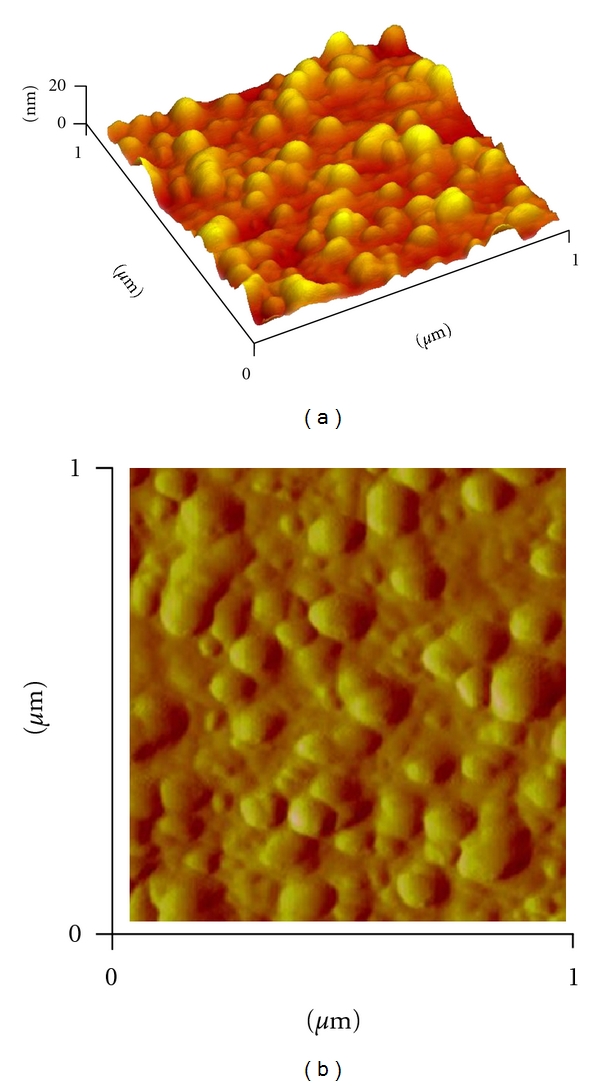
AFM images of biofunctionalized AgNPs embedded in organic moiety.

**Figure 6:**
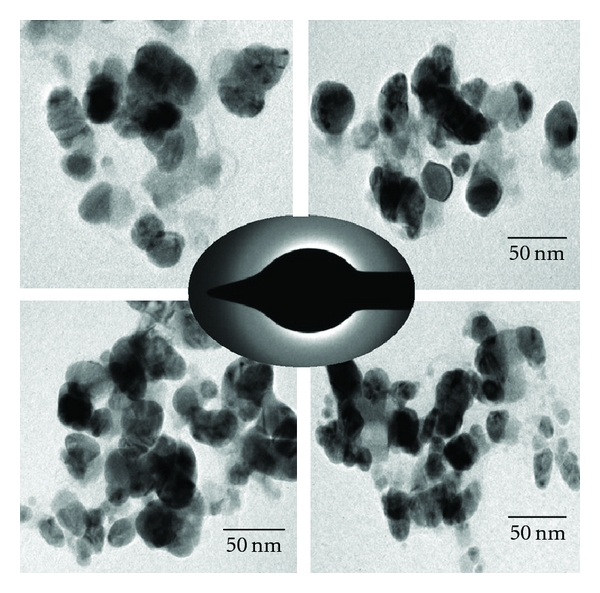
TEM images of AgNP.

 In order to verify the results of the UV-vis spectral studies, the colloidal suspensions of AgNPs was examined by XRD to confirm crystallinity.  Figure 3 shows that XRD pattern is obtained for biologically synthesized AgNPs. A number of Bragg reflections corresponding to the (111), (200), (220), and (311) sets of lattice planes are observed which may be indexed based on the FCC structures of silver (JCPDS files no. 03-0921). Thus, the crystalline nature of AgNPs formed is confirmed. The Energy dispersive X-ray analysis study (EDAX) shown in the Figure 4 reconfirmed that the particles formed are crystalline in nature and are indeed metallic AgNPs. The occurrences of carbon and oxygen peaks reveal the presence of covering organic moieties on the metallic nanoparticles. The appearance of “Cu” in figure is because of the copper grid base used for the analysis. At present, AFM is a powerful tool to study the morphology of biofunctionalized particles. The biofunctionalization of the AgNPs prepared by using mushroom extract is further confirmed by AFM measurements. The three-dimensional study of biofunctionalized nanoparticles were made on tapping mode technique developed especially for studying biofunctionalization.  Figure 5 shows the AgNPs biofunctionalized having organic layer which consists of lot of organic moieties at the surface. From this figure, we can predict that the shape of the nanoparticles is nearly spherical with some irregular shaped particles and is randomly distributed. The typical TEM image showing the size and morphology of AgNPs is given in Figure 6; the morphology of AgNP is apparently spherical. From the TEM micrograph it is observed that the AgNPs formed are in a size range of 20 ± 5 nm and polydispersed. The selected diffraction pattern from one of the silver nanoparticle, is shown in the inset of Figure 6, which suggests that the AgNP is apparently in amorphous condition since organic moiety is covered on it. The crystallinity can be achieved by repeated centrifugation and washings.

**Figure 7:**
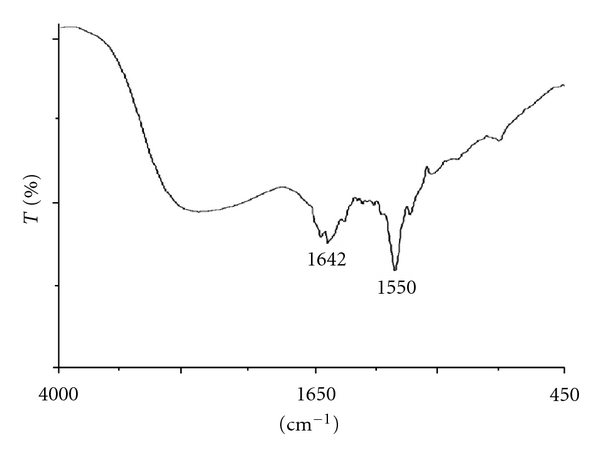
FTIR spectrum of AgNP.

FTIR spectroscopy from the absorption of IR radiation through resonance of noncentrosymmetric (IR active) modes of vibration is useful technique to study the core-shell morphology of AgNPs.  Figure 7 shows FTIR spectrum of AgNPs. The two bands at ~1642 and ~1550 are observed and are recognized as amide I and amide II and arise due to carbonyl stretch and –N–H stretch vibrations in the amide linkages of the protein correspondingly. IR spectroscopic study has confirmed that the carbonyl group from amino acid residue and peptides of proteins have stronger ability to bind metal, and so the proteins most possibly might have formed a layer on the AgNPs (i.e., biological capping) which also prevents agglomeration of the particles, and thus the nanoparticles are stabilized in the medium.

**Figure 8:**
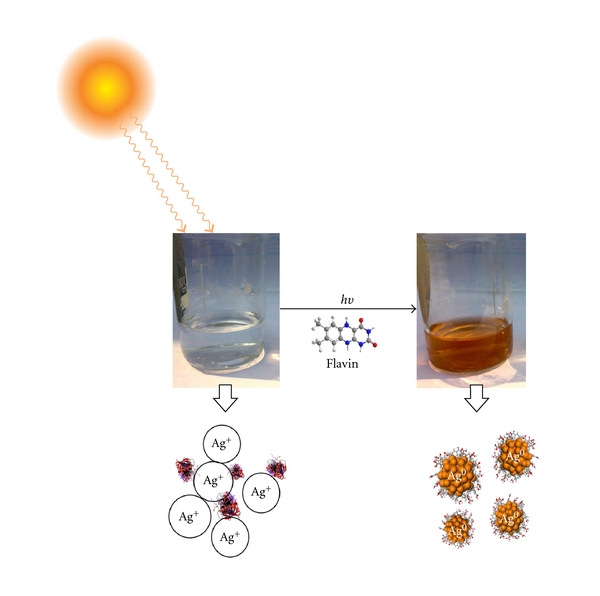
Probable pathway of synthesis mechanism.

Mushrooms are known for rich proteinacious food consisting of 75% proteins. Apart from proteins, Oyster mushrooms serve as an excellent source of riboflavin and other nutrients [35]. Riboflavin is water soluble and sensitive to light. Riboflavin functions in the bound coenzyme forms flavin mononucleotide (FMN) and flavin-adenine dinucleotide (FAD) as a catalyst for various reduction-oxidation reactions. Lumiflavin is the photoproduct of photo degradation of FAD, FMN, and riboflavin. Hence, it is understood that flavins (flavo proteins) present in the mushroom extract are responsible for the reduction of silver ion into AgNP, the reaction mixture when exposed to sunlight, absorbs photons of energy and the flavins present in the reaction mixture may get excited and become electron donors or oxidizers [36]. This is responsible for conversion of Ag^+^ to Ag^0^. The protein present is further believed to cap the AgNP formed, and thus they become functionalized. The figurative representation is shown in Figure 8.

 The exact mechanism of formation of the biofunctionalized nanoparticles in these biological extract is still not clearly understood. It appears that it is quiet probable that the flavin plays a vital role in the reduction of Ag ions to AgNPs as it is known for its photosensitivity and good oxidizing property.

The separation of flavin from the mushroom extract and to study its reduction mechanism is our next goal.

### 3.1. Antibacterial Mechanism

**Figure 9:**
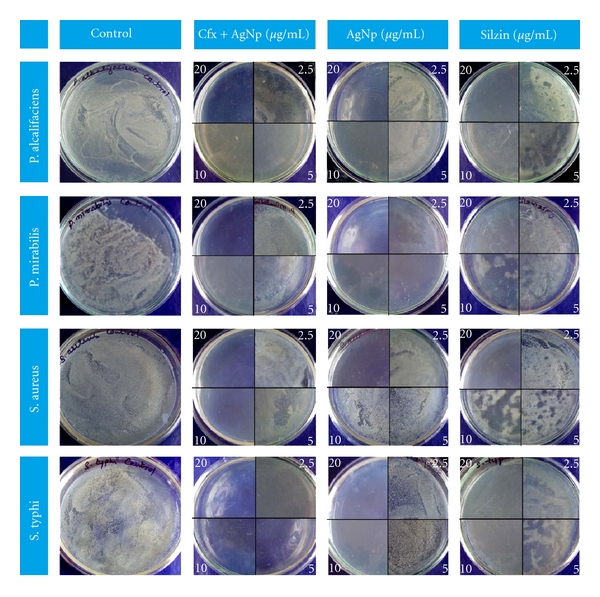
Antimicrobial activity of the AgNP using edible mushroom *Pleurotus florida*. Effects of AgNP with cefixime, AgNP, and the standard drug silver sulfadiazine (silzin) in different concentrations are shown in the figure.

To investigate growth inhibition effect of Ag-NPs solution against different microbes, namely, *Staphylococcus aureus, Salmonella typhi, Providencia alcalifaciens, *and* Proteus mirabilis*, we measured the MIC and the changes of bacterial counts at different concentrations of cfx + AgNP (equal quantities of cefixime antibiotic drug and functionalized silver nanoparticles), AgNPs (functionalized silver nanoparticles), and with silzin (standard silver sulfadiazine drug), (2.5 *μ*g to 20.0 *μ*g/mL concentration) shown in Figure 9. The control plates are prepared without addition of any drug for the comparative study of the inhibitory effects of the cfx + AgNP, AgNP, and silzin. These results exposed that the MIC of cfx + AgNPs showed higher-inhibition kinetics against both *P. alcalifaciens*, *P. mirabilis* (Gr +ve). *S. aureus*, and* S. typhi* (Gram −ve) when compared with other samples shown in Table 1. Only 2.5 *μ*g/mL cfx + AgNP is sufficient for the complete prevention of bacterial growth of *S. typhi*, whereas in case of other three bacteria 10.0 *μ*g/mL of cfx + AgNP is to be added for the growth inhibition. From the result, it is noted that AgNP synthesized from mushroom is showing decent bactericidal activity against gram +ve bacteria, specially in case of *P. mirabilis *2.5 *μ*g/mL is sufficient for complete effect for reduction of bacterial colony, and in case of gram −ve bacteria the antimicrobial activity of AgNP is moderate. When the MIC result of both the samples (cfx + AgNP and AgNP) is compared with standard silzin, it is observed that the MIC of silzin is poor and more quantity of samples is required for complete growth reduction of bacterial colony. So, it is clear that biofunctionalized AgNP shows excellent antimicrobial property either by mixing with antibiotic drug or by using directly as a drug.

 It is presumed that the influence of free electrons produced by the surface of the functionalized AgNPs will have lethal effect on the electronegative surface membrane of the microorganism causing its death. The outcome of antibiogram analysis of compounds against different bacteria shows that biologically prepared AgNP can become an alternative agent in antibacterial treatment, because of its less side effect and potential action.

## 4. Conclusion

In this study, AgNPs were synthesized by the photo-irradiation technique using the edible mushroom,* Pleurotus florida, *as a bioreductant. The biosynthetic method developed in this study for producing silver nanoparticles has distinct advantages over chemical methods such as high biosafety and being ecofriendly and nontoxic to the environment. Furthermore, these functionalized silver nanoparticles showed a noticeable antimicrobial activity against different clinically important pathogenic microorganisms. Hence, such type of synthesis methods for the production of nanostructured materials at lower cost and with natural energy may encourage production of functionalized AgNPs on industrial scale. This is quite handy for using AgNPs on a wide range of applications in the field of nanobiotechnology.

## Figures and Tables

**Table 1:** MICs of sample against different pathogenic bacteria.

Sample	MIC (*μ*g/mL)			
	*S. aureus *	*S. typhi*	*P. alcalifaciens*	*P. mirabilis*
Cfx + AgNP	10.0	2.5	10.0	10.0
AgNP	20.0	10.0	10.0	2.5
Silzin	20.0	20.0	20.0	20.0
